# A qualitative study in parental perceptions and understanding of SIDS-reduction guidance in a UK bi-cultural urban community

**DOI:** 10.1186/s12887-016-0560-7

**Published:** 2016-01-30

**Authors:** Denise Crane, Helen L. Ball

**Affiliations:** Department of Anthropology, Durham University, Dawson Building, South Road, Durham, DH1 3LE UK

**Keywords:** Sudden infant death syndrome, SIDS reduction, Infant care, Bi-cultural, Qualitative research

## Abstract

**Background:**

Differences in both Sudden Infant Death Syndrome (SIDS) rates and infant care practices between white British and South Asians in UK are well known, but research has not yet examined how these two groups understand and implement SIDS-reduction guidance. This study aimed to discover how white British and Pakistani mothers in Bradford recall, understand and interpret SIDS-reduction guidance, and to explore whether and how they implement this guidance in caring for their infants.

**Methods:**

In-depth narrative interviews with 46 mothers (25 white British origin and 21 Pakistani origin) of 8–12 week old infants recruited from the pool of participants enrolled in the ‘Born in Bradford’ (BiB) cohort study.

**Results:**

All mothers were aware of UK SIDS-reduction guidance from leaflets presented to them during antenatal or postnatal interactions with health care providers. Pakistani mothers tended to dismiss the guidance in toto as being irrelevant to their cultural practices; white British mothers dismissed, adapted and adopted aspects of the guidance to suit their preferred parenting decisions and personal circumstances. Many mothers misunderstood or misinterpreted the guidance given and explained their infant care behaviour according to their social and cultural circumstances.

**Conclusions:**

Current SIDS reduction information in the UK does not meet the needs of immigrant families, and is easily misinterpreted or misunderstood by mothers from all sections of the community. Tailored information acknowledging cultural differences in infant care practices is vital, as is greater discussion with all mothers about the reasons for SIDS reduction guidance.

**Electronic supplementary material:**

The online version of this article (doi:10.1186/s12887-016-0560-7) contains supplementary material, which is available to authorized users.

## Background

Unexplained sleep-related infant mortality (Sudden Infant Death Syndrome) is closely related to infant care practices and can vary substantially between immigrant and host populations [1–6]. Differences in infant care practices between white British and UK Pakistani mothers have been well documented, as has the much lower prevalence of Sudden Infant Death Syndrome (SIDS) of South Asian Infants in UK [7, 8]. The overall UK SIDS rate is currently 0.3/1000 live births, or approximately 250 infant deaths per year [9]. Current SIDS-reduction guidance is disseminated by health professionals (usually by means of leaflets often in multiple languages) at prenatal and postnatal contacts. Although it is known that South Asian infant care protects infants from the most important SIDS-related factors such as cigarette smoking, alcohol consumption, sofa-sharing, prone sleep and solitary sleep, research has not explored why parents appear to follow some guidelines and not others [7, 8].

The present study aimed to elicit detailed qualitative explanations for inter-cultural differences in infant care in the bi-cultural community of Bradford and explore the cultural context in which infant care is performed in order to inform future public health interventions in this and similar ethnically diverse populations.

## Methods

The consolidated criteria for reporting qualitative research (COREQ) are used to describe the study methods. COREQ provides a 32-item checklist for explicit and comprehensive reporting of qualitative studies (in-depth interviews and focus groups) [10].

We aimed to conduct 50 in-depth narrative interviews to obtain qualitative data from mothers of 8–12 week old infants (this age group is the peak age-range for SIDS in the UK). Recruitment was conducted at Bradford Royal Infirmary Maternity Unit when the mothers-to-be were 28 weeks pregnant and being recruited into the ‘Born in Bradford’ (BiB) cohort study. A purposive sample of 137 BiB participants were approached and asked if they would agree to be interviewed when their infants were 8–12 weeks old; although 69 agreed to be contacted 23 were lost from the sample due to changes in contact details or relocation outside of the Bradford area--therefore 25 white British and 21 Pakistani mothers (*n* = 46) were interviewed. Of the 21 Pakistani mothers that were interviewed 5 were first generation (born in Pakistan) and sixteen were second generation (born in the UK).

All interviews were conducted and analysed by the first author, a white British mature female researcher with training and expertise in qualitative interviewing, with guidance and supervision by the second author. Interviews were audio-taped and took place face-to-face at the participants’ homes and guided with the use of an Interview Schedule (Additional File 1). They were conducted in 4 equal batches from the two ethnic groups concurrently to ensure that developing themes were explored and cross-checked. The interviewer produced verbatim transcripts which were thematically analysed via a continuous comparative method as they were generated. Themes were discussed and agreed by both authors.

Ethical approval was granted by Durham University and the NHS National Research Ethics Committee of Yorkshire and the Humber. R&D Management approval was granted by Bradford Teaching Hospitals NHS Foundation Trust and Bradford Institute for Health Research. Participants received oral and written information about the study at recruitment and again prior to the interview when written consent was obtained.

## Results

Socio-demographic information was obtained from the BiB database and confirmed with participants at interview (see Table 1 below). The participants inhabited the same geographical areas of Bradford with 20/21 Pakistani mothers and 23/25 white British mothers residing in the socio-economically deprived inner-city.

**Table 1:** Characteristics of the Participants

	White British (*n* = 25)	Pakistani (*n* = 21)
Mean Age (Range)	27.2 (19–38)	28.5 (20–41)
Marital Status		
Married	15 (60 %)	19 (90 %)
Partnered	4 (16 %)	0
Single	6 (24 %)	2 (10 %)
First Time Mother	15 (60 %)	9 (43 %)
Educational Status		
Degree or Higher	3 (12 %)	1 (5 %)
A-level or equivalent	10 (40 %)	6 (29 %)
GCSE or equivalent	12 (48 %)	11 (52 %)
No qualifications	0	3 (14 %)
Employed	6 (24 %)	3 (14 %)

All participants knew about SIDS-reduction guidance, however, safe sleep practices were implemented variably (see Table 2 below). White British mothers acknowledged the recommendations to keep babies in their room for the first 6 months yet 7/25 were in separate bedrooms before 3 months of age: *“I really don’t want him to get used to being in our bedroom…I think the 6 month thing is just too old”; “She’s going to be in her own room soon. I don’t think she needs to be with me till she’s as old as 6 months.”* White British mothers also tended to place their infants in separate rooms for daytime sleep for quietness, ‘privacy’ and to allow mothers time to carry out chores. Pakistani mothers kept their babies in the parental bedroom at night and close to them during daytime sleep: *“I like him with me even if he’s sleeping. I know the English put their babies in their own rooms but Pakistani mums don’t do that.”; “Pakistani families always have the babies there…in daytime and in night. It’s just expected…it wouldn’t feel right if my babies weren’t with me.”*

**Table 2:** The Participants’ Infant Care Behaviours

	White British	Pakistani
Night-Time Sleep Location	Parents Room = 18 (72 %)	Parents Room = 21 (100 %)
	Own Room = 7 (28 %)	Own Room = 0
Infant Daytime Sleep Location	Separate Room = 16 (64 %)	Separate Room = 4 (19 %)
	Same Room = 9 (36 %)	Same Room = 17 (81 %)
Sleep Surface^a^	Cot/Crib/Moses	Cot/Crib/Moses
	basket/Bassinette = 23 (92 %)	basket/Bassinette = 13 (62 %)
	Bed Sharing = 2 (8 %)	Bed Sharing = 8 (38 %)
Sleep Bedding^b^	Sleeping bag = 17 (68 %)	Sleeping bag = 4 (19 %)
	Sheets/blankets = 8 (32 %)	Sheets/blankets = 17 (81 %)
	Pillow = 0	Pillow = 11 (52 %)
Sleep Position	Back = 17 (68 %)	Back = 21 (100 %)
	Back/side = 8 (32 %)	Back/side = 0
Sleep Surface Position^c^	Feet to Foot = 25 (100 %)	Feet to Foot = 9 (43 %)
	Top/middle = 0	Top/middle = 12 (57 %)
Sofa-Sharing	Yes (more than once) = 5 (20 %)	Yes (more than once) = 0
	Yes (once) = 3 (12 %)	Yes (once) = 0
	No = 17 (68 %)	No = 21 (100 %)
Infant Temperature Concerns	Hot = 23 (92 %)	Hot = 0
	Cold = 0	Cold = 14 (67 %)
	Both = 2 (8 %)	Both = 7 (33 %)
Use of Baby Intercom Systems	Yes = 18 (72 %)	Yes = 0
	No = 7 (28 %)	No = 21 (100 %)
Use of Infant Temperature Monitors	Yes = 25 (100 %)	Yes = 3 (14 %)
	No = 0	No = 18 (86 %)
Infant Feeding	Breast = 10 (40 %)	Breast = 8 (38 %)
	Formula = 15 (60 %)	Formula = 10 (48 %)
	Both = 0	Both = 3 (14 %)
Dummy (Pacifier) Use	Yes = 13 (52 %)	Yes = 3 (14 %)
	No = 12 (48 %)	No = 18 (86 %)

^a^Specific type of bed or mattress was not determined for bed sharing

^b^Sleeping bag is an infant night garment (gro-bag, sleep sack etc.)

^c^Feet to Foot is to position infants with their feet to the bottom of the crib or cot

All white British mothers were familiar with recommendations to place babies supine and ‘feet-to-foot’, although a third reported that sometimes infants slept on their side. Some mothers questioned whether infants could roll from the side to the prone position during sleep: *“I mean she’s too little to be able to get herself right over onto her tummy, isn’t she?”* All Pakistani mothers placed their babies supine for sleep but about half did not implement the feet-to-foot position due to the use of ‘infant pillows’. These items are not typical European-style pillows but thin infant head positioners that pose negligible suffocation or re-breathing risks due to their inability to mould around the infant’s face. The use of such ‘pillows’ was described as a valued tradition amongst Pakistani families and several mothers reported they were used against the explicit advice of health professionals: *“Even if they tell you not to use them, the mothers still use them anyway. I mean, they aren’t there 24/7 so the mothers just say they don’t use them and when they’re gone get them back out…it just saves the hassle.”*

Recommendations to avoid bed-sharing with an infant were known to the Pakistani mothers who expressed strong beliefs about this practice and dismissed guidance that babies should sleep in a cot near the parents’ bed, feeling this information was not directed to them: *“Most of the stuff they write is meant for the English mothers and not the Asian women. I know what’s best for my babies and I like them with me at night.”*

All white British mothers reported that they would avoid bed-sharing, implementing alternate options for feeding their babies during the night: *“I used to let her sleep with me cos she were feeding 2, 3 or even 4 times in night and I were really careful with her. I suppose I stopped cos I felt guilty, cos I know it’s not recommended, cos it’s a bit dangerous for them, but it was just so much easier for her to feed and for me.”* Although cautious of bed-sharing, a third of white British mothers reported sleeping on a sofa with their babies even while acknowledging the advice not to do so: *“I know they tell you not to but sometimes it’s hard when they’re up in night…I know I wouldn’t do it if I thought that I could hurt my baby so I was always careful.”* Some mothers avoided sofa-sharing by regularly using an armchair: *“I always just sit on a chair…I don’t sit on sofa cos I know it’s dangerous and it’s more possible to fall asleep on sofa.”* In contrast Pakistani mothers reported never sofa-sharing which was explained as being socially and culturally unacceptable in Pakistani households: *“The leaflets they give you say that you shouldn’t sleep on sofas with babies cos it’s dangerous but it’s just something the English do cos a respectful Asian woman just wouldn’t do that.”*

SIDS-reduction guidance emphasises the importance of preventing babies from overheating. Pakistani mothers were aware of this, but none were concerned about their infant becoming too hot: *“I don’t think the Pakistani mothers worry about the hot like the English do cos that’s always in the leaflets and stuff cos of cot death…They don’t ever mention anything about how being cold can make you poorly as well though do they? I suppose it’s more for the English.”* In contrast the white British mothers expressed concern about their babies becoming too hot due to the fear of SIDS and all used room temperature monitors for reassurance in contrast to Pakistani mothers who did not use temperature monitors.

Table 3 (below) displays the prevalence of cigarette smoking and alcohol consumption among the participants. All Pakistani mothers were aware of the link between maternal smoking and SIDS-risk although not of the association between paternal smoking and SIDS: *“I know mothers shouldn’t smoke and good Pakistani women don’t smoke. I don’t think it’s as important about fathers, is it?”* All of the white British mothers knew of the smoking and SIDS association but those who reported smoking modified their behaviour: *“I tried to stop but can’t. I never smoke near [baby]…I only smoke outside and never in here so it don’t affect her.”*

**Table 3:** Parental Smoking and Alcohol Consumption

	White British	Pakistani
Maternal Cigarette Smoking	3 (12 %)	0
Maternal Alcohol Consumption	15 (60 %)	0
Paternal Cigarette Smoking	2 (8 %)	4 (19 %)
Paternal Alcohol Consumption	17 (68 %)	0

Pakistani mothers and fathers reported no alcohol consumption and considered this advice as being for ‘English’ families: *“I don’t really think that stuff about smoking and drinking in them leaflets… it’s not for us…it’s aimed at the English mothers really isn’t it?”* White British mothers acknowledged the advice regarding alcohol consumption and SIDS but those who consumed alcohol reported they ‘only drank at weekends’ or ‘on special occasions’ and they ‘did not get drunk’. They did not feel that paternal alcohol consumption was relevant. Several white British mothers believed that advice regarding smoking and alcohol related to bed-sharing or breastfeeding mothers only: *“I know there’s risk with smoking and drinking when you have your baby in bed with you but I can’t see how smoking or a little drink can affect a baby when you’re not sleeping together…I don’t have my baby in bed with me and I don’t breastfeed now so it don’t affect us.”*

Overall none of the mothers interviewed followed all SIDS-reduction guidance and some implemented very little. While some Pakistani infant care practices corresponded with SIDS-reduction guidance this reflected cultural practice and was more coincidental than planned. White British mothers followed their personal preferences. The reasons supporting SIDS-reduction guidance were poorly understood.

## Discussion

Mothers living in the same neighbourhoods of a northern UK city, who had received and were aware of national SIDS-reduction guidance, implemented different infant sleeping practices for personal and cultural reasons. Many SIDS-reduction practices were part of normal Pakistani infant care (supine sleep; room-sharing; avoiding alcohol, smoking and sofa-sharing); as much of the guidance was irrelevant to Pakistani mothers they felt that Safe Sleep guidance was not directed to them. Consequently, where the guidance conflicted with traditional Pakistani beliefs about infant sleep (bed-sharing, pillow use, overheating) mothers prioritised their traditional practices and dismissed the official guidance. White British mothers consciously adapted or dismissed several aspects of the guidance (room-sharing; avoiding sofa-sharing) that they felt to be not applicable or unnecessary, while questioning others (lateral sleep position; any smoking or alcohol consumption). Their responses also suggested that the implementation of some SIDS-reduction practices compensated for the lack of others, highlighting parental perceptions that risk-reduction strategies can be traded against each other.

Growing evidence indicates that SIDS is less prevalent among infants who sleep near their parents [11–13]. However, keeping babies in the parental bedroom for 6 months was dismissed by many white British mothers who also placed their infants in separate rooms for daytime sleep: solitary sleep both at night and during the day was a common white British infant care practice. These participants were unaware that adult presence during daytime sleep is equally important as for night-time sleep [14, 15].

In previous research we found that Pakistani infants were significantly less likely than white British infants to sofa-share [7, 8]. In the present study Pakistani mothers explained that lying on a sofa was socially and culturally unacceptable, signifying laziness; Pakistani women remained in their bedrooms at night to feed their babies. White British mothers attempted to avoid bed-sharing by sofa-sharing, propping themselves up in bed, or using armchairs to feed their infants at night, even though SIDS-reduction guidelines includes avoidance of sofas and armchairs for night-time feeding. There is no specific advice to parents on how to feed babies safely during the night; this is an important omission that leaves mothers trying to choose between several apparently dangerous locations.

All white British mothers were aware of dangers in their baby’s sleeping environment; the majority did not use pillows or blankets preferring baby sleeping bags for night-time sleep. Pakistani mothers preferred sheets and blankets and infant ‘pillows’ (head positioners) even though they were aware of disapproval from health professionals regarding the latter. White British mothers were sceptical of the advice to avoid the side sleep position for infants, however, studies from England and New Zealand have shown that this position is unstable and infants risk rolling into the prone position [16–19]. Participants were unaware of this instability or that side sleeping infants could fall into the prone position before they were physically capable of rolling, suggesting some explanation is needed here.

Multiple studies have confirmed an association between maternal smoking, alcohol consumption and SIDS [20–23]. White British mothers’ attitudes to alcohol consumption warrants further research as some participants were unaware that alcohol could influence their infant care judgements and decisions (such as unintentionally falling asleep with a baby on a sofa). Furthermore, and importantly, many white British mothers believed smoking and alcohol consumption to be factors associated with SIDS only in conjunction with breastfeeding or bed-sharing, suggesting that these relationships may have been over emphasised.

Pakistani mothers repeatedly asserted that UK SIDS-reduction guidelines were not applicable to them noting that pictures and diagrams within the literature did not depict their own social and cultural environments, and the messages did not acknowledge or reflect their cultural beliefs and practices; this supports the view that SIDS-reduction messaging should acknowledge the culturally valued parenting behaviours and beliefs of the target communities in order to make messages more effective [24–26].

In summary, this study found that UK SIDS intervention strategies were limited in their effect on both Pakistani and white British families in Bradford as mothers were aware of, but did not implement, the risk-reduction guidance offered. Similar ineffectiveness has recently been reported in SIDS-reduction campaigns in north-west England [27] and Australia [28]. To date, safe sleep promotion and SIDS reduction has been founded on a ‘health persuasion’ model of risk elimination, described by Beattie as an authoritative top-down expert-led approach [29]. Given the well-known disparities in SIDS rates across ethnic, socio-economic and other sub-cultural groups, new approaches are clearly needed. One promising approach is to refocus SIDS reduction around Beattie’s ‘health negotiation’ model of risk minimisation involving tailored information delivered at community and individual levels [29]. We have discussed the need for risk-minimisation in infant safe sleep recommendations in more detail elsewhere [2–4], and the UK National Institute for Health and Care Excellence has recently recommended an individually tailored approach [30].

Additionally, we suggest that SIDS advice, literature and leaflets should depict the multi-cultural social and ecological contexts in which infant care is performed. Mothers may consider SIDS advice more readily if they see that the guidance acknowledges their cultural preferences. Health professionals could be trained to be more aware and understanding of how infant care decisions are mediated and performed amid differing social and cultural environments, and to provide tailored information to allow parents to work within their personal constraints to make informed choices regarding SIDS-risk reduction.

## Conclusion

UK SIDS rates have plateaued, and current public health interventions are not reducing them further. As both white British and UK Pakistani mothers resist adopting certain SIDS guidance according to their personal, social and cultural environments this suggests individually tailored approaches to SIDS-reduction would be more effective than the ‘single simple message’ approach.

One of the key findings in this study was that mothers felt they could ‘trade-off’ which components of SIDS guidance to implement, and that adhering to some of the recommendations (e.g. supine sleep) counteracted lack of adherence to others (e.g. smoking); the provision of evidence to counter this perception might help increase adherence to the most efficacious interventions. Therefore, it is suggested that more emphasis is placed on those recommendations that are associated with the greatest risk reduction in order to achieve the greatest benefit; this may require specific quantitative modelling for specific populations or communities.

The findings of this study suggest guidance and literature should be culturally tailored with acknowledgement, understanding and appreciation of variations in infant caregiving and discussion of the barriers to implementation encountered in different settings. Infant health messages could work with parents’ cultural values and beliefs more effectively to identify ways to promote infant health and safety for specific social and cultural environments.

## Additional file

Additional file 1:
**Interview Schedule.** (DOCX 16 kb)

## Abbreviations

BiB: born in Bradford
BradICS: Bradford Infant Care Study)
SIDS: sudden infant death syndrome)
