# Supplementary material for: A qualitative study in parental perceptions and understanding of SIDS-reduction guidance in a UK bi-cultural urban community
Source: BMC Pediatr. 2016 Jan 30;16:23. doi: 10.1186/s12887-016-0560-7 (PMC4736706; doi:10.1186/s12887-016-0560-7)
Supplement: Additional file 1: — Interview Schedule. (DOCX 16 kb) [file 12887_2016_560_MOESM1_ESM.docx]

**Interview Schedule**

Birth/Household/Feeding/Night Sleep environment

***Could you talk me through a typical day in the life of you and your baby?***

Feeding

- Did you breastfeed your baby and why or why not? If yes how long and why?
- When was any other introduced (formula, drinks, solids) and why? What does baby get now?
- Is baby fed at night? How often and in what way?

Baby’s Night Sleep

- What surface does baby sleep on?
- What room does baby sleep in (alone or not)?
- What position is baby put to sleep in and what position does baby wake up in?
- What does baby sleep with-pillow, duvet, gro-bag, hat?
- Is baby swaddled at night?
- Is the heating on in baby’s room (day and/or night)?
- Is window left open?

Bed Sharing or Sofa Sharing

- Does baby bed share with mother or anyone else?
- If yes, does baby sleep on or in the bed, edge or middle of bed, separate covers (how many layers), swaddled, anyone else in the bed?
- Does baby sofa share with mother or anyone else?

Baby’s daytime naps

- What is the sleep environment of baby’s daytime naps and why?

Day Care

- Who? When? How often?

Temperature

- More concerned about hot/cold/both/none and why? Room temperature monitor?

Dummy Use

- Does baby have a dummy? If so why, when and for how long? If not why?

Infant’s bath time

- How is this practiced – where/when/how often/by whom etc?

Daily Interactions

- Play and Stimulations – where/when/how often/by whom etc?

Household arrangements - are mothers being influenced by family members?

Have mothers knowingly departed from cultural norms? - Why or why not?

Establish how much attention mothers pay to health information - who do they listen to for advice?

Educational Status/Household Income/Smoking and Alcohol
